# The Role of PPAR-**γ** and Its Interaction with COX-2 in Pancreatic Cancer

**DOI:** 10.1155/2008/326915

**Published:** 2008-07-02

**Authors:** Guido Eibl

**Affiliations:** Hirshberg Laboratories for Pancreatic Cancer Research, Department of Surgery, David Geffen School of Medicine, University of California, Los Angeles, 675 Charles E. Young Drive South, MRL 2535, Los Angeles, CA 90095, USA

## Abstract

In recent years, the study of the peroxisome proliferators activated receptor gamma (PPAR-*γ*) as a potential target for cancer prevention and therapy has gained a strong interest. However, the overall biological significance of PPAR-*γ* in cancer development and progression is still controversial. While many reports documented antiproliferative effects in human cancer cell and animal models, several studies demonstrating potential tumor promoting actions of PPAR-*γ* ligands raised considerable concerns about the role of PPAR-*γ* in human cancers. Controversy also exists about the role of PPAR-*γ* in human pancreatic cancers. The current review summarizes the data about PPAR-*γ* in pancreatic cancer and highlights the biologically relevant interactions between the cyclooxygenase and PPAR system.

## 1. INTRODUCTION

 Despite advances in surgical techniques, imaging modalities, and intensive care, pancreatic cancer is still an almost universally lethal disease with annual mortality figures virtually equaling incidence numbers. An estimated number of 37 170 patients hse been diagnosed with pancreatic cancer in 2007, and 33 370 patients have succumbed to that disease in the same year [1]. Absence of specific symptoms, lack of early detection markers, aggressive tumor growth, and virtual resistance to conventional chemo- and radiotherapy conspire to culminate in a median overall survival of less than nine months. Currently, surgical removal of the tumor offers the only hope of long-term survival with 5-year survival rates approaching 25–30% in large-volume centers in the US [2]. Although an adjuvant treatment regimen after surgical resection seems to prolong survival, the precise treatment protocol including drug-of-choice is still debated and the focus of several ongoing clinical trials [3]. Only a disappointing 10–15% of patients at the time of diagnosis are candidates for surgical resection and even patients who have undergone “curative” resection often die of recurrent tumor. The majority of pancreatic cancer patients unfortunately present with locally advanced or metastatic tumors which render them ineligible for surgical resection. Gem-citabine, an S-phase nucleoside cytidine analog, has been the standard chemotherapeutic drug for locally advanced and metastatic pancreatic cancer for more than ten years, but the improvement of overall survival is unacceptably small, often approaching only a few weeks [4]. Currently, several trials are underway that investigate gemcitabine-based combination therapies in patients with advanced pancreatic cancers. Capecitabine, an oral fluoropyrimidine carbamate and 5-fluorouracil prodrug, and erlotinib, an inhibitor of the epidermal growth factor receptor, are two promising agents which seem to improve survival in combination with gemcitabine compared to gemcitabine monotherapy [4]. The encouraging results from a large, double-blind, placebo-controlled, international phase III trial led to the approval of erlotinib for the treatment of locally advanced and metastatic pancreatic cancer in combination with gemcitabine [5]. Although certainly noteworthy, the improvement of overall survival with the combination regimen, however, was only marginal compared to gemcitabine monotherapy [5], strongly emphasizing the need for the identification of novel targets and the development of more efficacious therapeutic agents.

Although several environmental risk factors for the development of pancreatic cancers, including tobacco smoking and dietary factors, have been described, detailed insights into the pathogenetic mechanisms are virtually lacking [6]. Dietary intake of high-caloric, high-fat diets with ensuing obesity and metabolic syndrome has been correlated with an increased risk of pancreatic cancer [7, 8]. An important molecule in fatty acid sensing and metabolism is the peroxisome proliferator activated receptor gamma (PPAR-*γ*), a member of the nuclear receptor superfamily that functions as a ligand-activated transcription factor [9]. There is now a large body of evidence demonstrating an important role of PPAR-*γ* in the metabolic syndrome [10–13]. The thiazolidinedione (TZD) class of PPAR-*γ* ligands has been used for the treatment of hyperglycemia and insulin resistance in type 2 diabetes for the past ten years [14]. In addition, TZDs may also show beneficial effects on cardiovascular complications associated with type 2 diabetes and the metabolic syndrome [14–17]. More recently, the role of PPAR-*γ* in various human cancers has been studied. There is now strong evidence that PPAR-*γ* is overexpressed in a variety of cancers, including colon, breast, prostate, stomach, lung, and pancreas [18–20]. However, the biological significance of PPAR-*γ* is still controversial [21, 22]. Although several reviews highlight the antiproliferative actions of PPAR-*γ* ligands in cell culture and animal models of human cancers [23, 24], more recent studies illustrating a tumor-promoting effect of PPAR-*γ*, in particular in colon and breast cancer models, raise considerable concern about the significance and safety of PPAR-*γ* ligands as anticancer drugs [25–29]. This review will summarize and discuss the data concerning the role of PPAR-*γ* in pancreatic cancer.

## 2. PPAR-GAMMA IN PANCREATIC CANCER

Reports from several groups have shown that the thiazolidinedione (TZD) class of PPAR-*γ* ligands attenuates the growth of pancreatic cancer cells in vitro by induction of terminal differentiation and G1 phase cell cycle arrest [30, 31], and by an increase in apoptotic cell death [32]. Furthermore, thiazolidinediones attenuated pancreatic cancer cell migration and invasion by modulation of actin organization and expression of matrix metalloproteinase-2 and plasminogen activator inhibitor-1, respectively [33, 34]. However, many growth-inhibitory effects of PPAR-*γ* ligands are independent of PPAR-*γ* [35]. To date, several non-PPAR-*γ* targets have been implicated in the antitumor activities of certain TZDs ,for example, troglitazone and ciglitazone, including intracellular Ca^2+^ stores, mitogen-activated protein kinases, cyclin-dependent kinase inhibitors p27kip1 and p21WAF/CIP1, the tumor suppressor protein p53, and Bcl-2 family members [36]. There is increasing evidence that TZDs directly affect mitochondrial function which impairs oxidative respiration leading to increased reactive oxygen species (ROS) production and ATP depletion, which in turn can activate AMP kinase [37]. An increase in ROS and activation of AMP kinase can lead to PPAR-*γ* independent reduction in inflammation and cell growth [37]. In addition, it has been shown in pancreatic cancer cells that 2-cyano-3,12-dioxooleana-1,9-dien-28-imidazolide (CDDO-Im), a partial PPAR-*γ* agonist, induces apoptosis directly by targeting mitochondrial glutathione [38]. Furthermore, 3,3′-diindolylmethane (DIM), another PPAR-*γ* agonist, induced apoptotic cell death in pancreatic cancer cells through activation of the endoplasmic stress response [39]. Overall, the potential to elicit PPAR-*γ*-independent effects may be ligand- and cell context-dependent. Our own studies have demonstrated that PPAR-*γ* is expressed in the nucleus of six human pancreatic cancer cells and that treatment of these cells in vitro with 15-deoxy-Δ^12,14^-prostaglandin J2 (15-PGJ2) and ciglitazone dose- and time-dependently decreases cell growth by induction of caspase-3-dependent apoptosis [40]. In addition to their antiproliferative actions, both ligands, 15-PGJ2 and ciglitazone, reduced the invasive capacity of pancreatic cancer cells in vitro by a PPAR-*γ*-mediated decrease of urokinase-type plasminogen activator and elevation of plasminogen activator inhibitor-1 expression that resulted in an overall reduction in urokinase activity [41]. Taken together, there is a strong evidence today from cell culture models that PPAR-*γ* ligands potently reduce the growth of human pancreatic cancer cells. The discrepancy of the reported underlying mechanisms, however, may be caused by the use of different cell lines, culture conditions, and experimental settings. In contrast to the notion of PPAR-*γ* ligands being potent antitumor drugs in pancreatic cancers, we have reported that treatment of human pancreatic cancer cells in vitro with 15-PGJ2 and troglitazone dose-dependently increases the secretion of the vascular endothelial growth factor (VEGF), which is widely recognized as a potent stimulus for tumor angiogenesis [42]. In addition, the culture medium of troglitazone-treated human pancreatic cancer cells enhanced migration of endothelial cells, another step in the angiogenic cascade (own finding). These findings are already observed at submicromolar concentrations of the PPAR-*γ* ligands, which are usually considerably lower than the typical ligand concentrations needed for the antiproliferative effects in pancreatic cancer cells. Our in vitro data suggest that PPAR-*γ* ligands may have a tumor-promoting effect in vivo by enhancing tumor angiogenesis. Although the precise role of PPAR-*γ* in tumor angiogenesis is still debated and controversial, there is accumulating evidence that activation of PPAR-*γ* stimulates VEGF production and neoangiogenesis also in other cell models [43, 44]. 

In addition to the effects of PPAR-*γ* ligands on the growth of established pancreatic cancers in preclinical cell culture and xenograft mouse models, dietary intake of 800 ppm pioglitazone for 22 weeks correlated with an improved serum lipid profile and a decreased incidence and multiplicity of pancreatic tumors in the N-nitrosobis(2-oxopropyl)amine (BOP) model of pancreatic carcinogenesis in Syrian golden hamsters, suggesting a potential chemopreventive role of TZDs [45]. 

There are very few data concerning the significance of PPAR-*γ* in clinical pancreatic cancer specimens. In a recent study, PPAR-*γ* was expressed in the majority of human pancreatic cancer specimens, positively correlated with higher tumor stage and grade, and interestingly was associated with shorter patient survival, suggesting a potential role in pancreatic cancer progression [20]. 

## 3. INTERACTION BETWEEN THE PPAR-GAMMA AND COX-2 PATHWAYS

**Figure 1:**
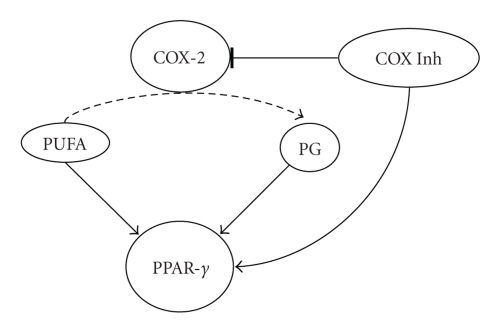
Possible interactions between the COX-2 and PPAR-*γ* pathways: polyunsaturated fatty acids (PUFAs) are substrates for cyclooxygenase-2 (COX-2) enzymes leading to the formation of various prostaglandins (PGs). Certain PUFAs and PGs can also activate PPAR-*γ*. Selective and nonselective COX-2 inhibitors (COX Inh) block PG formation by COX-2 but can also at higher concentrations activate PPAR-*γ*. Solid arrows indicate activation; dashed arrow indicates metabolic pathway; blocked arrow indicates inhibition.

Besides the TZD class of antidiabetic drugs, various intracellular lipids and lipid mediators are capable of activating PPAR-*γ*. Among those, polyunsaturated fatty acids (e.g., arachidonic acid (AA) and eicosapentaenoic acid (EPA)) and eicosanoids (e.g., 15-deoxy-Δ^12,14^-prostaglandin J_2_ (15-PGJ_2_)) are also substrates and products, respectively, of intracellular cyclooxygenase (COX) enzymes strongly suggesting relevant interactions between the PPAR and COX pathways (see Figure 1).

### 3.1. COX products as PPAR-*γ* activators

COX activity leads to the formation of an unstable hydroxy-endoperoxide, prostaglandin H_2_, which can be further converted to various prostanoid species by tissue specific isomerases [46]. While parent prostaglandins (e.g., PGE_2_, PGF_2*α*_, and PGD_2_) transduce their signals through binding to G-protein coupled cell surface receptors [47], cyclopentenone prostanoids (e.g., PGJ_2_) are known ligands of PPAR-*γ* [48]. In fact, there is evidence suggesting that COX-2 is preferentially located on the nuclear membrane allowing cyclopentenone prostaglandins to directly enter the nucleus and bind to ligand-activated transcription factors [49]. In this regard, human pancreatic cancer cells seem to express COX-2 preferentially in a perinuclear localization [50]. 15-deoxy-Δ^12,14^-prostaglandin J_2_ (15-PGJ_2_), a nonenzymatically formed dehydration product of PGD_2_, is detectable in COX-2 expressing human pancreatic cancer cells (own observation) and able to activate PPAR-*γ* in these cells [42]. Furthermore, a selective COX-2 inhibitor at a concentration that inhibits COX-2 activity and consequently prostanoid production reduces PPAR-*γ* activity, presumably by decreasing the levels of cyclopentenone prostaglandins (own observation).

### 3.2. COX substrates as PPAR-*γ* activators

Certain polyunsaturated fatty acids (PUFAs) (e.g., arachidonic acid (AA; 20 : 4 *n*−6) and eicosapentaenoic acid (EPA; 20 : 5 *n*−3)) are substrates for COX enzymes and also known PPAR-*γ* ligands [51]. Both PUFAs are released from the *sn-2* position of major membrane phospholipids by phospholipase A_2_ (PLA_2_) enzymes, particularly by the cytoplasmic PLA_2_, which upon activation seems to preferentially locate to the nuclear membrane [52, 53]. Once released, the PUFAs can be metabolized by COX enzymes or enter the nucleus to activate PPAR-*γ*. Our own studies demonstrated that EPA decreased the growth of human pancreatic cancer cells through COX-2 dependent and independent mechanisms (manuscript in press). The COX-2 independent mechanism involved activation of PPAR-*γ* by EPA as the growth-inhibitory effect of EPA was abolished by a pharmacological PPAR-*γ* antagonist. Furthermore, EPA and to a lesser extent AA can activate PPAR-*γ* transcriptional activity in human pancreatic cancer cells (own observation). This effect is less pronounced in pancreatic cancer cells that express COX-2 presumably because EPA is rapidly metabolized by COX-2 in these cells. The overall efficacy of PUFAs to activate PPAR-*γ* may therefore be dependent on the cellular expression and activity of COX-2.

### 3.3. COX inhibitors as PPAR-*γ* activators

In addition to COX-2 substrates and products, certain nonselective and selective COX-2 inhibitors have also been shown to activate PPAR-*γ* independent of their ability to inhibit COX-2 enzymatic activity [54], although the precise molecular mechanisms are still unknown. There is a compelling evidence today that the inducible COX-2 isoform plays an important role in pancreatic cancer development and growth and that selective COX-2 inhibitors may be efficacious for pancreatic cancer prevention and therapy [50]. Our own studies demonstrated that dietary intake of a selective COX-2 inhibitor delayed the progression of recognized pancreatic cancer precursor lesions in a genetically engineered mouse model of pancreatic cancer development [55]. Furthermore, a selective COX-2 inhibitor decreased the growth of COX-2 positive human pancreatic cancers in a xenograft mouse model by induction of apoptosis in cancer cells and by inhibition of tumor angiogenesis [42]. In contrast, the selective COX-2 inhibitor enhanced the growth of xenografted human pancreatic cancers that lacked or had very little COX-2 protein expression. This tumor-promoting effect was associated with an increase in intratumoral VEGF levels and tumor angiogenesis [42]. Additional studies showed that the tumor-enhancing effect of the selective COX-2 inhibitor in COX-2 negative or weakly COX-2 expressing human pancreatic cancers was abolished by GW9662, an irreversible pharmacological PPAR-*γ* antagonist, suggesting biologically important interactions between the COX-2 inhibitor and PPAR-*γ* [42]. Further studies demonstrating enhanced PPAR-*γ* binding activity in tumors that were treated with a selective COX-2 inhibitor confirmed that interaction [42]. The findings obtained in vivo were corroborated by in vitro experiments. Human pancreatic cancer cells treated with relatively high concentrations of selective COX-2 inhibitors showed an increased production and secretion of VEGF, which was inhibited by a pharmacological PPAR-*γ* antagonist and a dominant-negative PPAR-*γ* receptor [42]. Additionally, the selective COX-2 inhibitor at that concentration stimulated PPAR-*γ* transcriptional and DNA-binding activities [42]. These data clearly indicated that a biologically significant interaction between selective COX-2 inhibitors and PPAR-*γ* exists and that activation of PPAR-*γ* by these drugs may have detrimental, that is tumor-promoting, effects on pancreatic cancer growth. It is important to note that the tumor-promoting effects of selective COX-2 inhibitors were only observed at relatively high concentrations (much higher than needed to inhibit COX-2 enzymatic activity) in tumors that had no or only very little COX-2 expression [42]. Although the selective COX-2 inhibitor stimulated VEGF production by pancreatic cancer cells through a PPAR-*γ* mediated mechanism also in COX-2 expressing pancreatic cancers, the potential proangiogenic and tumor-promoting effect in COX-2 positive cancers was masked by a significant reduction of COX-2 generated proangiogenic and protumorigenic prostanoids [42]. 

## 4. CONCLUSION

While several in vitro studies demonstrate that PPAR-*γ* activation decreases pancreatic cancer cell growth, the finding that PPAR-*γ* ligands can stimulate VEGF production by pancreatic cancer cells raises serious concerns that PPAR-*γ* activation in vivo may lead to enhanced angiogenesis and tumor growth. Further detailed studies using pancreatic cancer animal models and specific PPAR-*γ* ligands are necessary to evaluate possible proangiogenic and protumorigenic properties of PPAR-*γ* activation in vivo. Unfortunately, information about the role of PPAR-*γ* in pancreatic carcinogenesis is almost nonexistent. The use of the recently developed genetically engineered mouse models of pancreatic cancer development that closely recapitulate our current knowledge of pancreatic cancer development on a histological and genetic level should shed some needed insights into the role of PPAR-*γ* in pancreatic carcinogenesis.

There is now clear evidence of a biologically relevant interaction between the COX and PPAR-*γ* pathways. Our data suggest that activation of PPAR-*γ* by selective and nonselective COX-2 inhibitors may have tumor-promoting effects in vivo by enhancing tumor angiogenesis. The effect of COX-2 inhibitors on PPAR-*γ* activation seems to be observed only at relatively high concentrations of the inhibitors and the overall biological phenotype of that interaction is dependent on the cellular expression and activity of the COX-2 protein. Although the role of PPAR-*γ* in pancreatic cancer development and growth has begun to be elucidated in recent years, a precise knowledge of molecular targets downstream of PPAR-*γ*, a more comprehensive elucidation of PPAR-*γ*-independent actions of PPAR-*γ* ligands, and a detailed understanding of crosstalks between PPAR-*γ* and other intracellular signaling pathways seem to be absolutely necessary and needed to eventually clarify the role of PPAR-*γ* in human cancer development and progression.
